# Immune checkpoint inhibitor-associated diabetes mellitus: mechanisms, clinical manifestations, and management strategies

**DOI:** 10.3389/fendo.2025.1679751

**Published:** 2026-01-13

**Authors:** Yu Chen, Xiaolu Wang, Shuyun Duan

**Affiliations:** 1 Department of General Practice, Hangzhou Third People’s Hospital, Hangzhou, Zhejiang,, China; 2 Department of General Practice, Hangzhou Third Hospital Affiliated to Zhejiang Chinese Medical University, Hangzhou, China

**Keywords:** autoimmunity, checkpoint inhibitor-associated diabetes mellitus, diabetic ketoacidosis, immune checkpoint inhibitors, PD-1, PD-L1, T cell, β-cell

## Abstract

Immune checkpoint inhibitor–associated diabetes mellitus (ICI-DM) is a rare but life-threatening endocrine immune-related adverse event characterized by abrupt insulin deficiency and a high incidence of diabetic ketoacidosis (DKA). Unlike classical type 1 diabetes, ICI-DM often develops after only a few treatment cycles, shows a fulminant phenotype with disproportionally modest HbA1c elevation, and is typically irreversible and glucocorticoid-refractory, necessitating permanent insulin therapy. Mechanistically, PD-1/PD-L1 blockade disrupts pancreatic immune tolerance and permits autoreactive CD8^+^ T-cell–mediated β-cell destruction, with risk amplified by susceptible HLA haplotypes and pre-existing islet autoantibodies (most commonly GAD antibodies). Additional contributors include pancreatic inflammation, cytokine-driven immune activation, incretin axis perturbations (GLP-1/GIP), and β-cell dedifferentiation programs. Clinically, timely recognition requires proactive glucose monitoring, early ketone assessment, and evaluation of C-peptide and pancreatic enzymes, particularly because DKA can occur rapidly after symptom onset. This review synthesizes current evidence on ICI-DM epidemiology, pathogenesis, clinical presentation, and management, and discusses emerging preventive and disease-modifying strategies, including mesenchymal stromal cells and B-cell–targeted approaches, to optimize outcomes in patients receiving immunotherapy.

## 1 Introduction

Immune checkpoint inhibitors (ICIs) have reshaped modern oncology by restoring antitumor immunity through blockade of key inhibitory pathways, most notably programmed death-1 (PD-1), programmed death-ligand 1 (PD-L1), and cytotoxic T-lymphocyte-associated antigen-4 (CTLA-4) (1). To date, the US Food and Drug Administration has approved 14 ICI agents, encompassing anti-PD-1 antibodies (pembrolizumab, nivolumab, sintilimab, toripalimab, camrelizumab, tislelizumab), anti-PD-L1 antibodies (atezolizumab, durvalumab, avelumab, envafolimab, sugemalimab), and the anti-CTLA-4 antibody ipilimumab (2). By design, these therapies amplify cytotoxic T-cell activity and broaden immune effector function, but this immune disinhibition also generates a distinctive spectrum of immune-related adverse events (irAEs) that differs fundamentally from toxicities observed with cytotoxic chemotherapy or molecularly targeted agents (3).

Among irAEs, endocrine toxicities are increasingly recognized as clinically relevant complications as ICI use expands across tumor types and treatment lines (3). Reported endocrine dysfunction affects approximately 30% of treated individuals and most commonly involves the pituitary, thyroid, pancreas, and adrenal cortex (4–6). Although pancreatic endocrine injury is uncommon, immune checkpoint inhibitor–associated diabetes mellitus (ICI-DM) is clinically disproportionate to its incidence because it often presents abruptly and may progress to fulminant diabetic ketoacidosis (DKA), a life-threatening emergency (7–10). Accordingly, timely recognition, mechanistic understanding, and standardized management are essential for optimizing outcomes. In this review, we synthesize current evidence on the epidemiology, clinical phenotype, pathogenesis, and management of ICI-DM, with the goal of providing a practical framework for surveillance and multidisciplinary care in routine oncology practice.

## 2 Epidemiology of ICI−DM

The 2022 American Diabetes Association classification recognizes immune checkpoint inhibitor–induced diabetes as an immune-mediated form of type 1 diabetes mellitus (T1DM) (11). Clinically, this phenotype closely resembles, and can be difficult to distinguish from, fulminant T1DM (12, 13). Across published cohorts, the reported incidence of ICI-DM ranges from 0.2% to 1.4%; however, its clinical impact is disproportionate to its frequency because more than half of patients present with DKA at diagnosis (8–10). Importantly, β-cell autoantibody positivity and combination regimens that co-blockade PD-1/PD-L1 and CTLA-4 are consistently associated with a higher likelihood of DKA, indicating that host autoimmunity and treatment intensity jointly shape disease severity (14, 15).

The risk of ICI-T1DM varies by therapeutic class, with the highest frequency observed under PD-1/PD-L1 plus CTLA-4 dual therapy, followed by PD-1 monotherapy, PD-L1 monotherapy, and CTLA-4 monotherapy (15). A plausible explanation is a “double-hit” mechanism: CTLA-4 blockade broadly expands and activates T cells, whereas PD-1 pathway blockade removes peripheral tolerance checkpoints that otherwise restrain β-cell–directed autoreactivity, thereby facilitating organ-specific immune injury (16). In contrast, CTLA-4 monotherapy rarely precipitates diabetes, potentially reflecting both narrower clinical utilization and the constitutive expression of CTLA-4 in pituitary tissue, which may bias toxicity toward hypophysitis rather than pancreatic autoimmunity (17). Consistently, agent-level estimates report T1DM incidences of 3.5% with nivolumab, 2.3% with pembrolizumab, 0.7% with atezolizumab, and 0.3% with durvalumab, whereas ipilimumab alone is only infrequently implicated (18, 19).

Epidemiologically, ICI-DM is most commonly reported in melanoma and non–small-cell lung cancer, likely reflecting both disease burden and patterns of ICI utilization (8). Demographic trends across case series indicate a male predominance (20–24). Across cohorts, median onset is approximately 7–17 weeks, typically after about four treatment cycles (25, 26). More broadly, the interval from ICI initiation to overt hyperglycaemia spans 1 week to 12 months, underscoring the need for ongoing vigilance throughout—and potentially beyond—the treatment course (27).

## 3 Clinical manifestations of ICI-DM

Immune checkpoint blockade can precipitate immune-mediated pancreatic injury; thus, ICI-DM is an acute insulin-deficient syndrome that often resembles T1DM. Patients may be asymptomatic or develop polydipsia, polyuria, and weight loss. Hyperglycaemia typically occurs from several days to 12 months after the first infusion and frequently presents with ketosis or overt DKA, while a minority show isolated hyperglycaemia. Onset is abrupt, and about half of patients develop DKA requiring intensive-care management (28–30). Consistent with rapid β-cell loss, endogenous insulin secretion is often exhausted within three weeks of clinical onset (31), and DKA tends to develop roughly two weeks after initial hyperglycaemic symptoms are recognized (32).

Notably, β-cell failure in ICI-DM generally proceeds more rapidly. Once pancreatic autoimmunity is triggered, islet function deteriorates swiftly, producing a phenotype that resembles fulminant T1DM (FT1DM) (33). FT1DM is characterized by marked hyperglycaemia despite disproportionately low glycated haemoglobin, minimal or absent C-peptide, and a high propensity for early ketosis or DKA (34, 35). Notably, approximately 43% of ICI-DM cases meet diagnostic criteria for FT1DM, a markedly higher proportion than that reported in acute-onset T1DM cohorts (36, 37). Autoantibody-positive patients appear to be at higher risk of DKA and display a fulminant course, with precipitous collapse of β-cell reserve (14). β-cell destruction in ICI-DM is slower than in classical FT1DM but substantially faster than in typical acute T1DM, supporting the concept of an intermediate yet highly aggressive autoimmune trajectory (32). Therefore, early recognition is essential for oncologists, endocrinologists, and emergency physicians. Although no deaths have been definitively attributed to ICI-T1DM, fatal events may be under-recognized or misattributed to malignancy rather than therapy-related toxicity. In contrast to FT1DM, where islet autoantibodies are usually absent, approximately half of patients with ICI-T1DM test positive for at least one islet autoantibody, reinforcing an immune-mediated pathogenesis. Concomitant immune-related adverse events (irAEs) are also common, occurring in nearly 40% of cases, most frequently thyroid dysfunction, with hypophysitis and adrenal insufficiency also reported (14). Accordingly, systematic evaluation for coexisting endocrine and non-endocrine irAEs is critical to reduce missed or delayed diagnoses and to guide comprehensive supportive care.

Laboratory features can be diagnostically informative. HbA1c often underestimates the severity and tempo of hyperglycaemia, remaining normal or only modestly elevated at presentation despite marked glucose derangements (10, 13, 20). Exocrine pancreatic involvement has also been reported. A meta-analysis found that approximately half of ICI-treated recipients develop elevations in pancreatic enzymes, including asymptomatic hyperenzymemia in 3% and overt pancreatitis in 2% (31). However, whereas most patients with FT1DM exhibit increased lipase, amylase, or elastase-1, many individuals with ICI-T1DM do not, suggesting partial dissociation between endocrine failure and overt exocrine inflammation in this setting. Circulating glucagon levels are generally normal (10). Imaging findings are heterogeneous, patients may exhibit pancreatic atrophy (10), pancreatic enlargement, or features consistent with diffuse pancreatitis (38). Pancreatic enlargement may precede diabetes onset and can be followed by progressive pancreatic volume loss (8). Taken together, these observations underscore the need for vigilant metabolic surveillance during ICI therapy and for rapid initiation of insulin replacement and standardized DKA management protocols when hyperglycaemia is detected.

## 4 Pathophysiological mechanisms of ICI-DM

### 4.1 PD−1/PD−L1 signaling and ICI-DM

ICI-DM is strongly associated with disruption of the PD-1/PD-L1 axis. PD-1 is an inhibitory receptor expressed on T cells, B cells, activated monocytes, and dendritic cells, and it binds PD-L1, which is broadly expressed across leukocytes, lymphoid organs, and parenchymal tissues, including pancreatic islets. Notably, β cells from individuals with type 1 diabetes or with islet autoantibody positivity exhibit upregulated PD-L1 expression, consistent with an inducible tissue-protective checkpoint response (39). In non-obese diabetic (NOD) mice, anti-PD-1 therapy precipitates rapid-onset diabetes (40), and genetic ablation of PD-1 or PD-L1 markedly accelerates disease onset (41). Antigen-specific tolerance models similarly demonstrate that PD-1/PD-L1 blockade is sufficient to provoke autoimmune diabetes (42). Mechanistically, interferon-γ enhances β-cell PD-L1 expression in NOD islets (39), whereas loss of PD-1 on CD4^+^ T cells augment islet antigen–specific infiltration, underscoring the pathway’s role in restraining diabetogenic autoimmunity (43).

Physiologically, PD-1 signaling limits T-cell activation and proliferation, and tumors exploit this inhibitory checkpoint to evade immune elimination; CTLA-4, expressed on naïve and regulatory T cells, competes with the co-stimulatory receptor CD28 for CD80/86 binding, thereby attenuating early T-cell priming. Therapeutic antibodies targeting PD-1, PD-L1, or CTLA-4 restore T-cell effector function and promote tumor cytolysis, but the resulting immune hyperactivation also underpins immune-related adverse events (44). In the pancreas, activated T cells can destroy β cells and thereby abrogate insulin production. PD-L1 expression localizes to islets containing β cells and correlates with CD8^+^ T-cell infiltration; concordantly, whole-blood RNA profiling from newly diagnosed ICI-DM cases demonstrates PD-L1 upregulation (45). In NOD models, PD-1/PD-L1 blockade reliably triggers diabetes, whereas transgenic overexpression of either PD-1 or PD-L1 reduces incidence or delays onset (46–49). Importantly, these studies indicate preferential upregulation of PD-L1—but not CTLA-4 ligands CD80/86—on β cells, supporting a dominant role for the PD-1 pathway in islet immune tolerance (50). Human mechanistic evidence remains limited; however, in a 63-year-old man with well-controlled type 2 diabetes treated with nivolumab for renal cell carcinoma with pancreatic metastasis, histology from non-tumorous pancreas revealed dense T-cell infiltration dominated by CD8^+^ over CD4^+^ cells, with few B cells or macrophages, implicating CD8^+^ T cells as principal effectors in CIADM pathogenesis (51). Collectively, findings from animal models and the emerging human data support the concept that PD-1/PD-L1 blockade permits the survival and expansion of autoreactive T cells, culminating in β-cell eradication and the onset of ICI-T1DM (52, 53).

### 4.2 Susceptibility genes/HLA polymorphisms and ICI-DM

The contribution of heritable factors to conventional T1DM is well established. HLA genotyping in selected T1DM cohorts has consistently shown that diabetes-predisposing HLA configurations are markedly over-represented compared with the general population (26). Approximately 90% of patients carry the DR3-DQ2 or DR4-D08 haplotypes (54, 55), with DR3-D02 and DR4-D08 single haplotypes comprising the principal risk alleles (56). In contrast, DR4-D04 and DR9-D09 haplotypes have been associated with fulminant diabetes in East-Asian populations (57). These observations provide a rationale for considering pre-treatment assessment of high-risk HLA alleles in patients scheduled to receive ICIs, with the goal of estimating the likelihood of ICI-T1DM and informing surveillance intensity.

Beyond classical HLA risk, emerging genomic data suggest that additional susceptibility loci may modulate ICI-DM pathogenesis. Comprehensive genomic analyses have identified 15 genes with elevated expression in ICI-DM (58). The most frequently reported coding alteration is a missense variant in NLRC5; additional missense variants in DNAJB11, PXN, and XRCC3 (58). Variants in NLRC5 and CEMIP2 appear particularly enriched in CIADM (58). Mechanistically, NLRC5 acts as a key transactivator of HLA class I genes, and its dysregulation may increase islet immunogenicity and thereby amplify autoimmune responses in predisposed individuals (59). This concept is biologically plausible given that the highly polymorphic HLA region on chromosome 6 is strongly linked to autoimmunity, and that class I and class II alleles are essential for CD8^+^ and CD4^+^ T-cell activation, respectively (60). The association of class II–restricted alleles with organ-specific autoimmunity further emphasizes the tissue-selective nature of HLA-mediated risk (60). Several studies have examined HLA haplotypes specifically in ICI-DM. In a retrospective series of 200 patients, HLA typing was available for 78 individuals; among these, 51% carried an HLA-DR4 haplotype, reported as a higher prevalence than that observed in Caucasian classical T1DM, whereas 14% carried HLA-DR3 and 10% possessed putatively protective haplotypes (61).

### 4.3 Islet autoantibodies and ICI-DM

T1DM is initiated by T cell–mediated destruction of pancreatic β cells, culminating in progressive insulin deficiency (62). In parallel, B cells contribute to disease initiation and risk stratification through the production of islet-directed autoantibodies (63). Approximately 95% of patients with classical T1DM test positive for at least one pancreatic autoantibody (63), and the presence of two or more distinct autoantibodies is associated with an estimated 50% risk of developing clinical T1DM (64). In the context of ICI-T1DM, pancreatic autoantibody positivity is observed in a substantial subset of patients and is widely regarded as a supportive biomarker of immune-mediated β-cell injury. Clotman et al. (26) reported that, among 39 patients with ICI-T1DM, 22 (56%) were positive for pancreatic autoantibodies, and all seropositive cases had glutamic acid decarboxylase autoantibodies (GADA). Additional autoantibodies included protein tyrosine phosphatase IA-2A, islet cell antibodies (ICA), insulin autoantibodies (IAA), and ZnT8A (26). Across cohorts, approximately 40% of patients with ICI-DM harbor classic T1DM-associated autoantibodies, with GADA being the most frequently detected specificity (65). Clinically, autoantibody positivity has been associated with a more rapid disease onset (66). For example, GADA-positive patients have been reported to develop ICI-DM after an average of three treatment cycles, whereas GADA-negative patients develop disease after approximately six cycles on average (67). Moreover, compared with patients lacking T1DM-related autoantibodies, seropositive individuals show a higher incidence of diabetic ketoacidosis (68).

### 4.4 T−cell subsets and ICI-DM

T cells play a crucial role in autoimmune diseases; however, their involvement in ICI-DM remains poorly understood. Research by Lozano et al. (69) demonstrated that the enrichment of activated memory CD4^+^ T cells in peripheral blood prior to ICI treatment, along with TCR diversity, is associated with the occurrence of irAEs. Existing autoimmune diseases or preclinical autoimmune states may serve as indicators for the potential development of severe irAEs. Previous studies have suggested a link between CD8^+^ T cells and latent autoimmune diabetes in adults (LADA). Furthermore, autopsy studies of ICI-DM patients have revealed marked infiltration of T lymphocytes in the pancreatic islets, with CD8^+^ T cells being more prominent than CD24^+^ B cells (70), further suggesting a potential association between CD8^+^ T cells and the development of ICI-DM. Additionally, other T helper subsets, such as Th17 and Th2, may also contribute to the pathogenesis of ICI-DM (71).

### 4.5 Glucagon-like peptide-1/gastric inhibitory polypeptide and CIADM

GIP and GLP-1 are intestinal peptides that enhance insulin secretion during meals and play a critical role in glucose homeostasis. Changes in their secretion have been linked to the development of abnormal glucose metabolism. Studies have shown that alterations in gut microbiota diversity or imbalances in key microbial species can affect enteroendocrine cell function, consequently influencing blood glucose levels in patients (72). A study focusing on the intestinal endocrine system in CIADM patients treated with ICIs demonstrated reduced GLP-1 levels in these patients. Additionally, GIP concentrations were found to be lower both during fasting and in oral glucose tolerance tests (OGTT) (73). These findings suggest that ICIs may influence gut microbiota, thereby altering enteroendocrine cell function and leading to decreased GLP-1 and GIP levels, which may contribute to the development of CIADM.

### 4.6 Pancreatic inflammation/β−cell dedifferentiation and ICI-DM

Many CIADM patients exhibit elevated serum amylase and/or lipase at, or preceding, diabetes onset, suggesting pancreatitis and subsequent pancreatic atrophy (74, 75). Biopsy specimens show peri−islet inflammation with interferon−γ and tumor−necrosis−factor−α expression. In PD−L1–blocked diabetic mice, transcriptomic analyses highlight enrichment of interferon−γ response genes, alongside β−cell dedifferentiation characterized by reduced Ins1/2, Chga, Mafa and Nkx6−1, and increased Gcg, Sst and Cxcl9 expression (76). Thus, cellular and humoral immunity, genetic polymorphisms, pancreatic inflammation and β−cell dedifferentiation collectively orchestrate CIADM pathogenesis. Continuous monitoring of islet autoantibodies and lipase may therefore improve early detection and severity assessment.

## 5 Therapeutic strategies of ICI-DM

**Figure 1:**
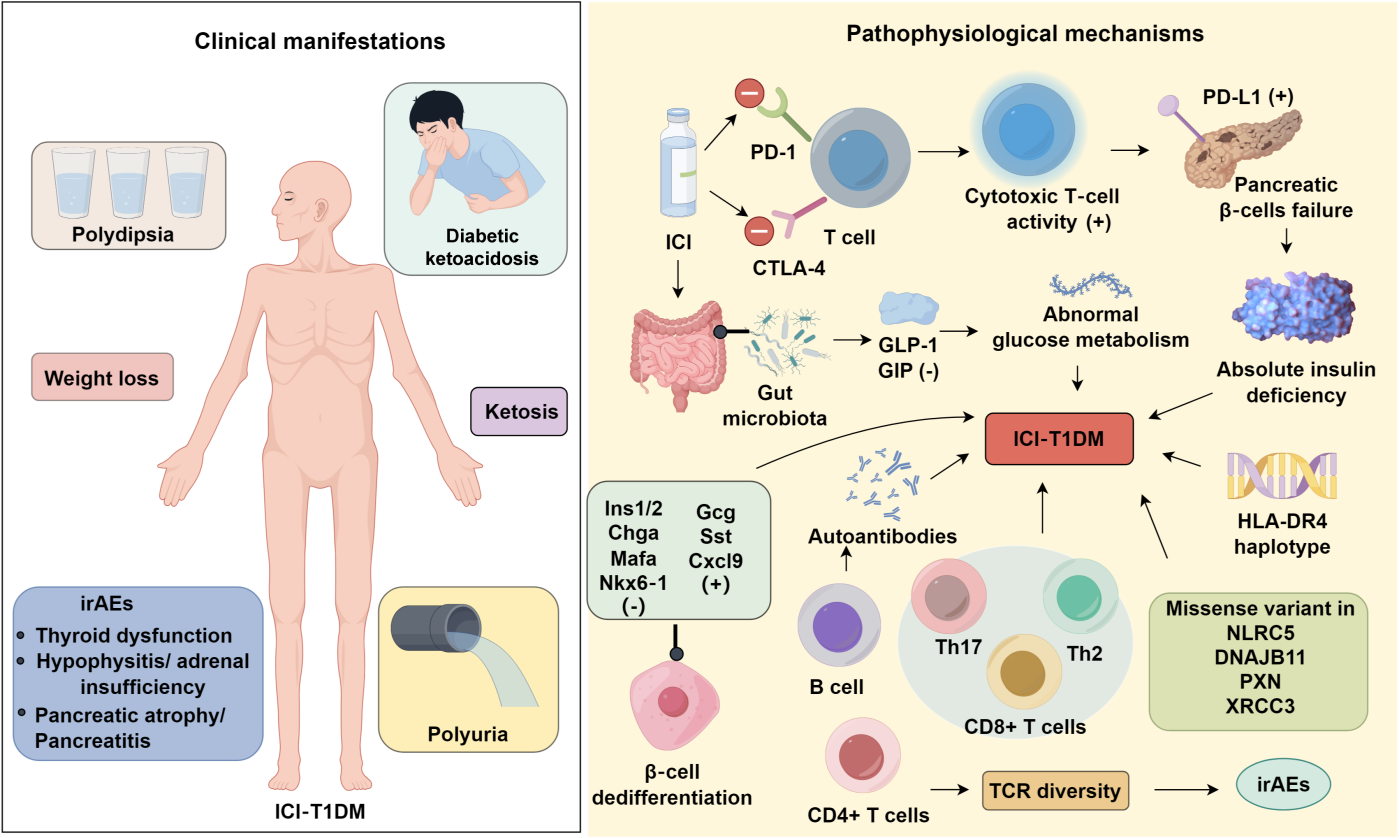
Immune checkpoint inhibitor-associated diabetes mellitus.

ICI-DM often progresses rapidly once it develops. After a definitive diagnosis, immediate initiation of exogenous insulin is strongly recommended, and oncologists should proactively counsel patients regarding the risk of ICI-DM and the high likelihood of permanent insulin dependence. When diabetic ketoacidosis occurs, urgent implementation of standard critical-care protocols—including aggressive intravenous fluid resuscitation, careful electrolyte correction, and continuous intravenous insulin infusion—is essential. Glucocorticoids are the first-line treatment for most immune-related adverse events; accordingly, multiple groups have attempted steroid therapy in ICI-DM (77–79). However, these interventions have been consistently ineffective. In contrast to many other irAEs, ICI-DM is generally irreversible and steroid-refractory (6); therefore, long-term glycaemic control typically relies on sustained insulin replacement (80). Beyond supportive management, emerging disease-modifying strategies are being explored. Mesenchymal stromal cells (MSCs), the most extensively studied cellular therapy platform, secrete bioactive mediators that promote tissue repair, limit fibrosis, and modulate immune responses. In male NOD mice, autologous MSC administration significantly suppressed PD-L1–associated diabetes onset (81). Quantitative histology demonstrated a larger residual β-cell area in MSC-treated animals, with pancreatic insulin content 1.9-fold higher than in untreated controls. MSC therapy also reduced islet-infiltrating T cells by 45% and CXCL9-positive macrophages by 67%, supporting a protective effect through attenuation of immune cell recruitment and tissue infiltration (81). In parallel, rituximab—a chimeric monoclonal antibody targeting the B-cell surface antigen CD20—has demonstrated the ability to delay T1DM progression in clinical trials (82) (**Table 1**). **Figure 1**.

**Table 1:** Pathogenetic mechanisms and emerging therapeutic strategies in ICI-DM.

Pathogenic mechanism	Key fndings	Potential therapeutic targets
PD-1/PD-L1 Disruption	PD-L1 upregulation on β-cells; CD8+ T-cell infiltration dominates;	Selective PD-L1 agonists (preclinical)
Genetic Susceptibility	HLA-DR4-DQ8 (51.3%); NLRC5/DNAJB11 variants enriched GADA+ (56%);	Pre-ICI HLA screening
Autoantibodies	IA-2A, ZnT8A less common; linked to shorter onset	B-cell depletion (rituximab)
T-Cell Subsets	Activated memory CD4+ T cells predict irAEs; Th17/Th2 involvement	TCR diversity modulation
Enteroendocrine Dysfunction	Reduced GLP-1/GIP levels post-ICI; gut microbiota alterations implicated	GLP-1 receptor agonists (experimental)
β-Cell Dedifferentiation	Downregulation of *Ins1/2*, *Mafa*; upregulated *Gcg*, *Cxcl9* in murine models	Mesenchymal stromal cells (MSCs; 67% macrophage reduction in mice)
Inflammatory Mediators	IFN-γ/TNF-α-driven peri-islet inflammation; elevated CXCL9	JAK/STAT inhibitors (theoretical)

## 6 Conclusion

Immune checkpoint inhibitor–associated diabetes mellitus (ICI-DM) is an uncommon yet potentially fatal immune-related adverse event that is increasingly encountered as ICIs are deployed across tumor types. In contrast to classical type 1 diabetes, ICI-DM typically presents with an abrupt transition from euglycaemia to profound insulin deficiency, frequently culminating in diabetic ketoacidosis and requiring intensive acute care. Current evidence supports a dominant role for PD-1/PD-L1 blockade in dismantling pancreatic immune tolerance and unleashing autoreactive, β-cell–directed cytotoxic T-cell responses, with disease kinetics shaped by host susceptibility factors such as high-risk HLA haplotypes, pre-existing islet autoantibodies, and permissive inflammatory programs within the pancreas. The emerging picture also implicates contributory mechanisms, including exocrine pancreatic inflammation, cytokine-driven immune activation, incretin axis perturbation, and β-cell dedifferentiation, that may collectively accelerate islet failure and account for the fulminant clinical phenotype.

From a clinical standpoint, early recognition and standardized management are paramount. Given the high incidence of ketosis/DKA and the frequent dissociation between marked hyperglycaemia and modest HbA1c elevations, routine glucose surveillance during ICI therapy, rapid assessment of ketones and C-peptide at symptom onset, and evaluation for concurrent endocrine irAEs are essential. Importantly, ICI-DM is characteristically refractory to glucocorticoids and is irreversible in most cases, making lifelong insulin replacement the mainstay of treatment and patient education a critical component of care. Looking forward, risk-adapted monitoring strategies incorporating HLA typing, islet autoantibodies, and pancreatic enzyme dynamics may enable earlier detection and mitigation. Therapeutically, adjunctive approaches aimed at preserving β-cell mass or tempering immune infiltration, such as mesenchymal stromal cells or B-cell–directed therapies, are biologically plausible and warrant prospective evaluation. Ultimately, improving outcomes will require multidisciplinary pathways that integrate oncology and endocrinology, alongside mechanistic studies to define actionable biomarkers and prevention-oriented interventions.
